# Stabilizing Enzymes in Plasmonic Silk Film for Synergistic Therapy of In Situ SERS Identified Bacteria

**DOI:** 10.1002/advs.202104576

**Published:** 2022-01-06

**Authors:** Zhangkun Liu, Shengkai Li, Zhiwei Yin, Zhaotian Zhu, Long Chen, Weihong Tan, Zhuo Chen

**Affiliations:** ^1^ Molecular Science and Biomedicine Laboratory State Key Laboratory of Chemo/Biosensing and Chemometrics College of Chemistry and Chemical Engineering College of Biology Aptamer Engineering Center of Hunan Province Hunan University Changsha 410082 China; ^2^ Faculty of Science and Technology University of Macau Taipa Macau 999078 China; ^3^ The Cancer Hospital of the University of Chinese Academy of Sciences (Zhejiang Cancer Hospital) Institute of Basic Medicine and Cancer Chinese Academy of Sciences Hangzhou 310022 China

**Keywords:** enzyme stabilization, graphitic nanocapsule, SERS, silk film, synergistic therapy

## Abstract

Increasing antibiotic resistance becomes a serious threat to public health. Photothermal therapy (PTT) and antibacterial enzyme‐based therapy are promising nonresistant strategies for efficiently killing drug‐resistant bacteria. However, the poor thermostability of enzymes in PTT hinders their synergistic therapy. Herein, antibacterial glucose oxidase (GOx) is embedded in a Ag graphitic nanocapsule (Ag@G) arrayed silk film to fabricate a GOx‐synergistic PTT system (named silk‐GOx‐Ag@G, SGA). The SGA system can stabilize GOx by a vitrification process through the restriction of hydrogen bond and rigid *β*‐sheet, and keep the antibacterial activity in the hyperthermal PTT environment. Moreover, the arrayed Ag@G possesses excellent chemical stability due to the protection of graphitic shell, providing stable plasmonic effect for integrating PTT and surface enhanced Raman scattering (SERS) analysis even in the GOx‐produced H_2_O_2_ environment. With in situ SERS identification of bacterial intrinsic signals in the mouse wound model, such SGA realizes superior synergistic antibacterial effect on the infected *Escherichia coli*, *Staphylococcus aureus*, and methicillin‐resistant *Staphylococcus aureus* in vivo, while without causing significant biotoxicity. This system provides a therapeutic method with low resistance and in situ diagnosis capability for efficiently eliminating bacteria.

## Graphical abstract

Antibacterial glucose oxidase embedded Ag graphitic nanocapsule arrayed silk film‐based efficient enzyme‐synergistic photothermal therapy (PTT) system is rationally designed. Such a versatile system can stabilize the antibacterial activity of enzymes in the hyperthermal PTT environment, while keeping the stable plasmonic effect for integrating PTT and in situ bacterial surface enhanced Raman scattering (SERS) identification in the enzyme‐produced H_2_O_2_ environment in vivo.

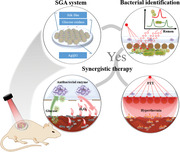

## 1 Introduction

Drug‐resistant bacteria infection has become one of the biggest problems threatening human health.^[^ ^1^ ^]^ Many treatment strategies for bacterial infections have been developed to address this tricky problem.^[^ ^1^, ^2^ ^]^ Photothermal therapy (PTT), as a nonresistant, rapid and effective therapeutic method with great potential for combination therapy,^[^ ^3^ ^]^ has attracted numerous efforts and possesses tremendous potential for drug‐resistant bacteria elimination. PTT makes use of the plasmonic property of noble metal nanoparticles, such as Au, Ag and Cu, or the calorigenic property of polymers by absorbing light to create a hyperthermia environment.^[^ ^4^ ^]^ The hyperthermia environment can cause irreversible oxidation of phospholipid layer of bacterial membrane, protein denaturation, cytoplasmic pyknosis and destruction of membrane structure, while without causing drug resistance.^[^ ^5^ ^]^ High temperature of ≈85 °C is required to realize an antibacterial efficacy of 90 %,^[^ ^6^ ^]^ and the excessive temperature can cause normal cell necrosis and pro‐inflammatory responses.^[^ ^7^ ^]^ To minimize these adverse effects, PTT combining plasmonic Ag nanoparticles (AgNPs) or antibiotics‐based synergistic strategies are subsequently developed. However, these synergistic PTT strategies still carry the risk of drug resistance^[^ ^8^ ^]^ or releasing of silver ions from AgNPs,^[^ ^9^ ^]^ resulting in unstable PTT effect and undesirable cytotoxicity. Therefore, developing antibiotic‐free, stable nonresistant synergistic method in PTT to minimize adverse effects of PTT is urgently needed.

Natural antibacterial enzymes, with low propensity to develop bacterial resistance,^[^ ^10^ ^]^ can attack pathogenic bacteria in diverse pathways, and exhibit great potential in synergistic PTT of bacteria. Among antibacterial enzymes, glucose oxidase (GOx) can continuously consume O_2_ and catalyze the oxidation of *β*‐D‐glucose into H_2_O_2_ and gluconic acid to realize the antibacterial effect.^[^ ^11^ ^]^ The produced H_2_O_2_ can cause the peroxidation of cell membranes, the oxidation of oxygen scavengers, thiol groups and nucleosides, and the disruption of energy production, enzyme function and protein synthesis.^[^ ^12^ ^]^ Meanwhile, the oxygen deficiency and lowered pH also affect bacterial growth negatively.^[^ ^3^, ^10^ ^]^ Moreover, with the continuous attacking of H_2_O_2_, the pathogenic bacteria could be sensitized to heat.^[^ ^13^ ^]^ Hence, antibacterial enzyme‐based therapy has great potential as the nonresistant synergistic method along with PTT to reduce adverse effects. However, the poor thermostability of antibacterial enzymes cause irreversible unfolding of the tertiary structure to a disordered polypeptide,^[^ ^14^ ^]^ further seriously hindering their practical application in the hyperthermia environment of PTT.^[^ ^15^ ^]^ Thus, stabilizing antibacterial enzymes in hyperthermia environment is the key for efficient and nonresistant enzyme‐synergistic PTT.

Among the enzyme stabilization methods against hyperthermia, physical embedding is rapid, simple and low‐cost comparing to enzyme engineering, chemical modification, crosslinking immobilization methods.^[^ ^14^, ^15^ ^]^ The choice of embedding material is critical for the enzyme stabilization. Regenerated silk fibroin (RSF), the main dissociated component of domesticated silkworm cocoons, stands out among different embedding materials in biomedicine field owing to its excellent biodegradability and non‐immunogenic nature.^[^ ^16^ ^]^ The versatile RSF can be manufactured into different geometries,^[^ ^17^ ^]^ and show an excellent performance of stabilizing drugs, antibodies, etc.^[^ ^18^ ^]^ Based on the stabilization in RSF materials, the thermostability of antibacterial enzymes can be significantly increased through physical embedding method,^[^ ^15a^ ^]^ which brings a new opportunity for the synergistic application of nonresistant bactericidal GOx along with the hyperthermal PTT process.

Herein, we rationally designed and fabricated a GOx‐synergistic PTT system (SGA) for bacterial elimination. The SGA consisted of GOx‐embedded silk film with arrayed plasmonic Ag graphitic nanocapsules (Ag@G). Such plasmonic SGA system could stabilize the embedded antibacterial GOx even in the hyperthermal PTT process to realize a continuous GOx‐synergistic PTT. The Ag@G, with Ag nanoparticle encapsuled in thin graphitic shell, possessed excellent chemical stability, providing stable plasmonic effect for PTT in H_2_O_2_ environment. Moreover, such Ag@G possessed excellent surface enhanced Raman scattering (SERS) effect while the SGA system was optical transparent because of the vitrification of silk fibroin.^[^ ^19^ ^]^ The transparent plasmonic SGA could provide the possibility of in situ bacterial SERS identification for guiding the GOx‐synergistic PTT.^[^ ^20^ ^]^ The SGA system demonstrated efficient GOx‐synergistic PTT effect on *Escherichia coli* (*E. coli*), *Staphylococcus aureus* (*S. aureus*), and methicillin‐resistant *Staphylococcus aureus* (MRSA) in vivo under the guideline of in situ bacterial SERS identification. This GOx‐synergistic PTT system provided an alternative method of stabilizing synergistic antibacterial enzymes in hyperthermal PTT for efficiently eliminating bacterial infection.

## 2 Results and Discussion

**Figure 1:**
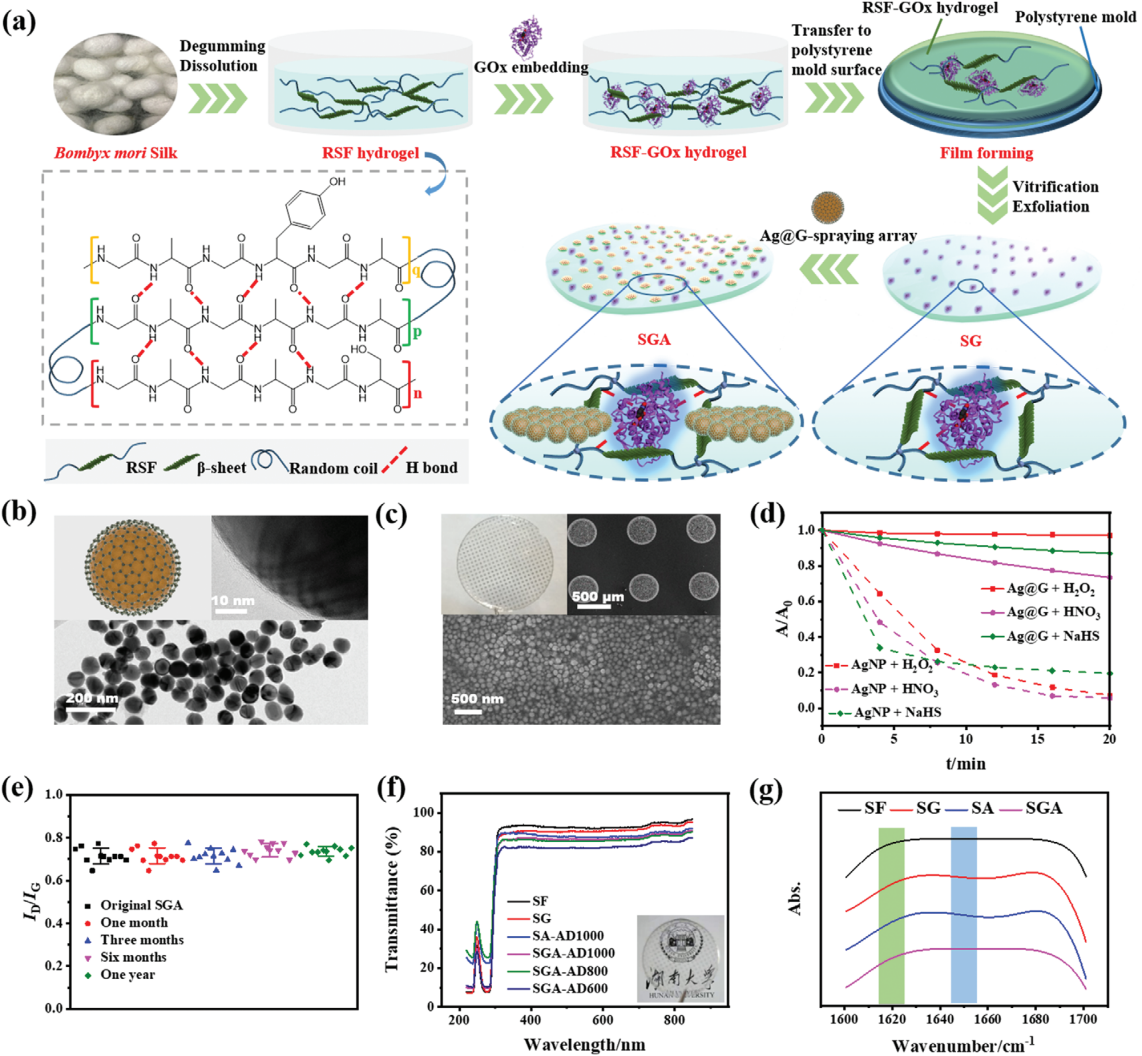
Construction and characterization of SGA. a) Schematic diagram illustrating brief construction process of SGA. b) TEM images of Ag@G. Inset: illustration (left) and high‐resolution TEM image (right) of Ag@G. c) SEM images of Ag@G on SGA. Inset: photograph of SGA (left) and Ag@G array on SGA (right). d) Chemical stability of Ag@G in H_2_O_2_, HNO_3_, and NaHS. e) SERS intensity ratio *I*_D_/*I*_G_ of SGA stored at room temperature for various times. f) Transmittance spectra of SGA with different distances of Ag@G array. Inset: photograph showing the transparency of SGA‐D1000. g) FTIR spectra of SF, SG, SA, and SGA.

The fabrication process of SGA was shown in **Figure** **1**a. First, RSF hydrogel, mainly consisted of hexapeptides of glycine (G), alanine (A), tyrosine (Y), and serine (S), was prepared through a degumming, dissociation and dialysis process. These hexapeptides typically included GAGAGS, GAGAGA, and GAGYGA.^[^ ^21^ ^]^ Then RSF hydrogel was mixed with antibacterial enzyme GOx. The RSF hydrogel and mixed RSF‐GOx hydrogel were dried to a pure silk film (SF) and a GOx‐embedded SF (SG) on polystyrene molds through a vitrification process, respectively. After Ag@G array sprayed on SF and SG respectively, transparent plasmonic Ag@G‐arrayed SF (SA) and Ag@G‐arrayed SG (SGA) integrating in situ Raman diagnosis with GOx‐synergistic PTT capabilities were obtained.

The ultraviolet–visible (UV–vis) absorption spectrum of Ag@G contained graphitic and Ag plasmonic absorption peaks at 265 and 435 nm, respectively (Figure S1a, Supporting Information). The plasmonic absorption peak extended to near infrared region, suggesting that Ag@G‐arrayed SGA could exhibit photothermal effect under 808 nm laser irradiation. The Ag@G demonstrated an average hydrodynamic diameter of ≈70 nm (Figure S1b, Supporting Information), and zeta potential of around ‐21 mV (Figure S1c, Supporting Information). The Ag@G was also characterized by transmission electron microscopy (TEM) (Figure 1b), the result verified a core‐shell Ag@G with ≈70 nm Ag core encapsuled in ≈1.6 nm graphitic shell. The enlarged TEM image of Ag@G with core‐shell structure was demonstrated in Figure S2 in the Supporting Information. The XRD spectrum (Figure S3a, Supporting Information) of Ag@G showed four peaks at 38°, 44°, 64°, and 77°, which corresponded to the crystal faces of (111), (200), (220), and (311) of face‐centered cubic crystal structure respectively. The peak of graphite was not obvious, which may be caused by the low degree of crystallization of the graphitic shell. The graphitic shell was characterized by Raman spectrum (Figure S3b, Supporting Information), which demonstrated graphitic (G) and disordered carbon (D) band at 1590 and 1330 cm^−1^, respectively. Microstructures of *Bombyx mori* silk (Figure S4a, Supporting Information) and degummed silk (Figure S4b, Supporting Information) appeared as smooth, flat ribbons under scanning electron microscopy (SEM). SEM images of SF surface (Figure S4c, Supporting Information) and section (Figure S4d, Supporting Information) exhibited a ≈100 µm‐thick smooth film. The line space of sprayed Ag@G arrays on SF was controlled by the microarray dot system. As shown in Figure 1c, the array of Ag@G dots was orderly and the size was uniform. The chemical stability of Ag@G was also investigated (Figure 1d). The Ag@G exhibited more robust chemical stability against 200 × 10^−3^ m H_2_O_2_, 400 × 10^−3^ m HNO_3_, and 2 × 10^−3^ m NaHS than bare AgNPs, suggesting Ag@G could be a stable Raman diagnosis and PTT platform even in H_2_O_2_ environment.^[^ ^22^ ^]^ Raman spectra of SGA (Figure S5, Supporting Information) contained G and D band, originating from the graphitic shell of Ag@G. The intensity ratio of D and G bands (*I*_D_/*I*_G_) in Figure 1e showed no significant increase, indicating that the Ag@G could maintain good quality for at least one year. To meet the requirements of in situ Raman diagnosis on wounds, the optical transparency of SGA was important. The transmittance spectra of SF, SG, SA, and SGA with different array distances (e.g., AD‐600, 600 µm in line space) all demonstrated an over 80% transmittance in the range of 380–850 nm (Figure 1f), which could be attributed to the optical transparency of SF/SG and the thin layers of Ag@G. In subsequent experiments, SGA‐AD1000 was chosen because of better transmittance than that of SGA‐AD600 and SGA‐AD800.

The protein secondary structures of SF, SG, SA, and SGA were also characterized by Fourier transform infrared (FTIR) spectroscopy (Figure 1g; Figure S6, Supporting Information). In amide I region (1600–1700 cm^−1^), 1620 cm^−1^ (green region), and 1698 cm^−1^ peaks were assigned to *β*‐sheet, 1645–1655 cm^−1^ peak (blue region) to random coil/helix, and 1685 cm^−1^ peak to *β*‐turn.^[^ ^23^ ^]^ The absorbance ratios of *β*‐sheet to random coil/helix were calculated to be 0.876, 0.815, 0.814, 0.760 for SF, SG, SA, and SGA, respectively. The results showed a reduced content ratio of *β*‐sheet of SG, SA, and SGA, suggesting the interaction of fibroin with embedded GOx and sprayed Ag@G disturbed the formation of *β*‐sheet. Further mechanical property was assessed on tensile tester (Figure S7, Supporting Information), SG, SA, and SGA were found to bear more strain but less stress than SF. The results suggested the reduced content of *β*‐sheet, since the strength and stiffness were correlated with the number of *β*‐sheet crystallites and the toughness was related to the amorphous matrix.^[^ ^21b^ ^]^ The results might originate from the interaction of embedded GOx with fibroin by hydrogen bond or the restriction of embedded GOx between rigid *β*‐sheet crystallites, which were beneficial to the stabilization of GOx.^[^ ^24^ ^]^ The hydrophilic performances of SF, SA, SG, and SGA were measured by the contact angle tests (Figure S8, Supporting Information). The results exhibited their hydrophilic surfaces with contact angles below 90° while these films combined hydrophobic repetitive domains of *β*‐sheet with hydrophilic non‐repetitive domains.^[^ ^25^ ^]^ The hydrophilic films could beneficial to cell proliferation and differentiation on wounds, which indicated the superior biocompatibility of the SGA.^[^ ^26^ ^]^

**Figure 2:**
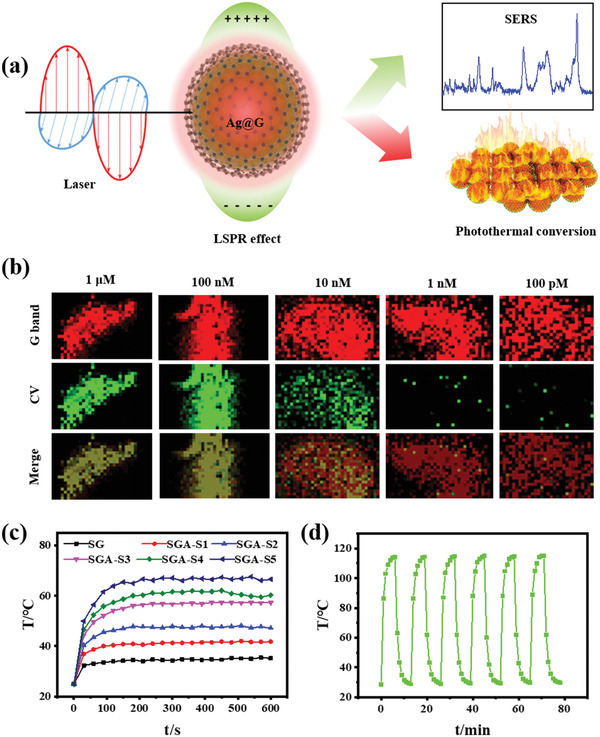
Plasmonic property of SGA. a) Schematic diagram of SERS and photothermal effect of Ag@G. b) SERS imaging of disinfectant CV in vitro by SGA. Pixel: 2 µm × 2 µm. c) Photothermal conversion capability of SGA with various Ag@G spraying doses (2500 mg L^−1^ Ag@G) under a 3 W cm^−2^ 808 nm laser. d) Photothermal stability test of SGA under a 5 W cm^−2^ 808 nm laser.

SGA possessed excellent photothermal conversion capability due to the localized surface plasmon resonance (LSPR) effect of Ag@G, which endowed it with huge potential for in situ bacteria Raman identification and effective photothermal therapy. The schematic diagram of SERS and photothermal effect of Ag@G was shown in **Figure** **2**a. First, the SERS effects of Ag@G on crystal violet (CV, a disinfectant, Figure S9, Supporting Information), and dipicolinic acid (DPA, a metabolite of bacterial spore, Figure S10, Supporting Information) were studied. The results indicated ≈3000‐fold and ≈11 000‐fold Raman signal enhancement for DPA and CV, respectively, indicating the superior SERS effect of Ag@G. Raman imaging of CV molecules was also implemented through the SGA to demonstrate the Raman diagnosis capability. The Raman imaging results demonstrated an ultra‐low detection limit of ≈100 × 10^−12^ m (Figure 2b). Then, photothermal conversion capability of SGA was investigated (Figure 2c). As the spraying amount of Ag@G increased, the film could reach a photothermal temperature over 60 °C under a 3 W cm^−2^ 808 nm laser. In order to demonstrate the thermal stability of SGA, a higher laser power of 5 W cm^−2^ was employed to achieve a photothermal temperature over 110 °C (Figure 2d). After six cycles of photothermal process, there was no obvious attenuation of photothermal conversion effect of SGA, which indicated the excellent thermal stability of SGA at photothermal antibacterial temperature.

**Figure 3:**
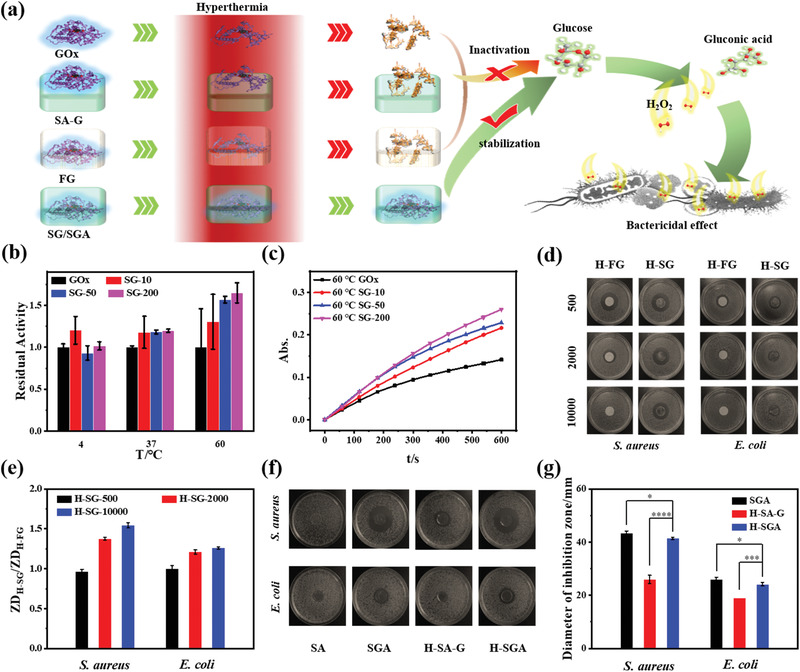
Stabilization of GOx by SGA in hyperthermia environment. a) Schematic diagram of the stabilization of GOx. b) Residual activity of GOx stabilized in SF with various RSF/GOx weight ratios (10, 50, and 200) at 4, 37, and 60 °C, respectively. c) Catalytic kinetics of GOx stabilized in SF with various ratios at 60 °C. d) Photographs and e) quantitative analysis of antibacterial effect of GOx stabilized in FP (FG) and SF (SG) with various ratios after 60 °C treatment for 10 d. f) Photographs and g) quantitative analysis of antibacterial effect of SA, SGA, H‐SA‐G, and H‐SGA. Error bar represents mean ± s.d., *n* = 3, ***p < 0.001, **p < 0.01, *p < 0.05 (two‐tailed *t*‐test).

PTT was generally combined with other antibacterial methods to reduce potential side effects of PTT.^[^ ^27^ ^]^ Antibacterial enzyme GOx had great potential to be employed in synergistic PTT by consuming glucose and O_2_ from wounds and producing H_2_O_2_. However, the GOx had a poor thermal stability in the hyperthermia environment.^[^ ^28^ ^]^ Encouragingly, the fibroin‐based SGA could stabilize the activity of GOx by hydrogen bonding and hydrophobic interactions.^[^ ^18a^ ^]^ Schematic diagram of stabilizing antibacterial GOx by SGA in hyperthermal environment was shown in **Figure** **3**a. Briefly, free GOx and GOx stabilized by different methods were subjected to a hyperthermia treatment. Then, the GOx activities of different groups were tested by monitoring the amounts and the antibacterial effects of produced H_2_O_2_. Before investigating the stabilizing effect of SF on the GOx, the enzyme‐like activity of Ag@G was tested (Figure S11, Supporting Information). With the same substrate, the Ag@G showed negligible peroxidase‐like activity comparing to the horse radish peroxidase (HRP). Comparing to the GOx, the Ag@G showed no glucose oxidation effect. Then, the activities of free GOx, and SG with different RSF/GOx weight ratios (10, 50, 200) were investigated after the treatment of different temperatures (Figure 3b,c; Figure S12, Supporting Information). Comparing to the free GOx treated at the same temperature, the activity of the embedded GOx was generally higher. More fibroin in the SG could achieve better GOx stabilization effect due to stronger interaction between GOx and fibroin. Filter paper (FP), usually used as the carrier in antimicrobial susceptibility test (AST), was employed to load GOx as the control group (FG). After heating FG (H‐FG) and SF (H‐SG) with different weight ratios under 60 °C, these H‐FG and H‐SG were subjected to the AST. To explore the superior GOx stabilization effect of SF, higher RSF/GOx weight ratios (500, 2000, 10 000) with lower concentrations of GOx were utilized (Figure 3d). Comparing to H‐FG groups, H‐SG groups all demonstrated higher antibacterial GOx enzyme activity. After normalizing the bacteria inhibition zone diameters of H‐FG (ZD_H‐FG_) and H‐SG (ZD_H‐SG_) in Figure 3e, the GOx stabilization effect of SF was obvious. Moreover, the H‐SG group also demonstrated higher antibacterial activity than H‐FG at room temperature (Figure S13, Supporting Information). The increased ratio of ZD_H‐SG_/ZD_H‐FG_ along with the increased RSF/GOx weight ratio revealed a better stabilization of GOx with more fibroin. The RSF/GOx weight ratio of 2000 (1 U, 4.425 µg GOx per film), demonstrating obvious difference of inhibition zone between H‐SG and H‐FG, was employed in the subsequent experiment. In special PTT process, the temperature need to reach around 85 °C.^[^ ^6^ ^]^ Hence, the condition of 85 °C photothermal treatment by 808 nm laser irradiation for 1 h was utilized to further investigate the stabilization of GOx (Figure 3f). As one control group, the SA showed no obvious inhibition zone, indicating that the SA containing SF and Ag@G had almost no antibacterial effect. The SA‐G was produced by coating GOx on the surface of SA. The H‐SA‐G and H‐SGA were produced by administrating SA‐G and SGA to 85 °C photothermal treatment, respectively. Comparing to the H‐SA‐G group, the H‐SGA group demonstrated higher antibacterial enzyme activity, verifying the superior GOx stabilization effect of SGA under PTT process. Through statistical analysis of inhibition zone diameters of SGA, H‐SGA and H‐SA‐G in Figure 3g, the H‐SGA group showed much higher antibacterial activity than that of the H‐SA‐G group, and showed just slightly reduced antibacterial activity comparing to the SGA group. The enzyme kinetics tests (Figure S14, Supporting Information) of GOx, SG, SGA, H‐SGA, and H‐SA‐G were employed to explain the difference in antibacterial effect. Michaelis constant *K_m_* was calculated from the Lineweaver‐Burk plots and equation

(1) $$\frac{1}{v}=\frac{K_{m}}{V_{max}}\;\cdot \frac{1}{\left[S\right]}+\frac{1}{V_{max}}$$

The *K_m_* values of GOx, SG, SGA, H‐SGA and H‐SA‐G were 0.0059, 0.0087, 0.0109, 0.0124, and 0.2175 mol L^−1^. Comparing to the SG, there is a negligible increase in the *K_m_* value of SGA, indicating that the Ag@G had little tendency to affect the activity of GOx in SF. Comparing to the SGA, the *K_m_* value of H‐SGA group showed little increase, while the *K_m_* value of H‐SA‐G greatly increased, which demonstrate that the GOx on H‐SA‐G without stabilization was inactivated and that the GOx stabilized in H‐SGA was still robust. Such evidences proved that the antibacterial GOx in SGA could be stabilized for hyperthermia resistance and play a stable synergistic bactericidal role in the hyperthermal environment of PTT process.

**Figure 4:**
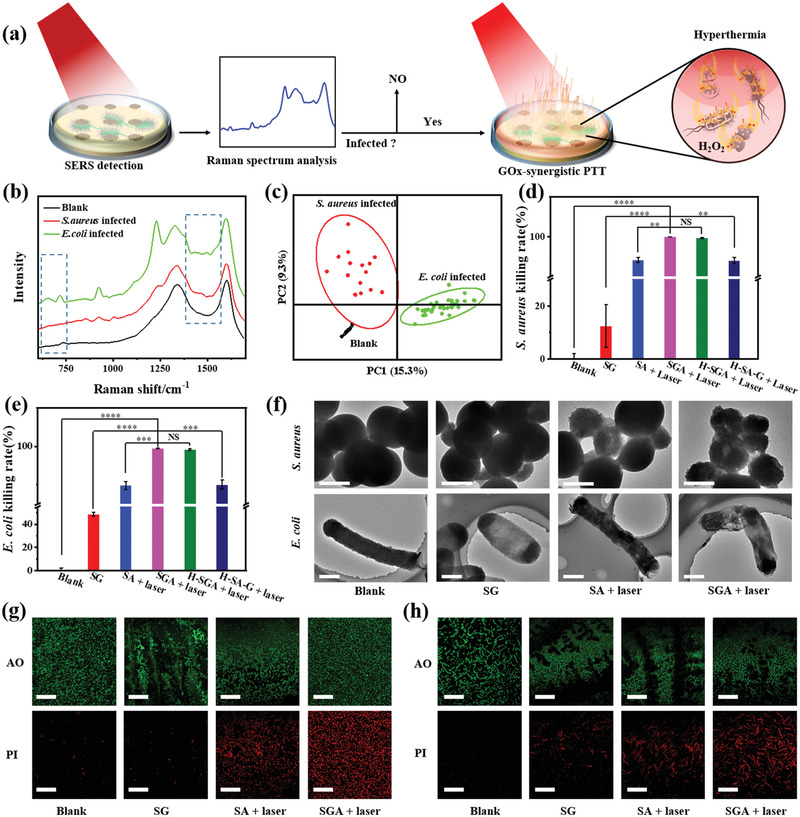
GOx‐synergistic PTT of bacterial infections in vitro. a) Schematic diagram illustrating GOx‐synergistic PTT of bacterial infections in vitro after SERS identification. b) Difference in SERS fingerprint spectra of *S. aureus* and *E. coli* in vitro. c) PCA identification of *S. aureus* and *E. coli* infections in vitro. d,e) Quantification of bacterial colonies after different treatments for *S. aureus* and *E. coli* in vitro. Error bar represents mean ± s.d., *n* = 3, ***p < 0.001, **p < 0.01, *p < 0.05 (two‐tailed *t*‐test). NS, not significantly different. f) TEM images of *S. aureus* and *E. coli* in different treatment groups. Scale bar: 500 nm. LSCM images of g) *S. aureus* and h) *E. coli* in different treatment groups. Green fluorescent acridine orange (AO) stained all cells, while red fluorescent propidium iodide (PI) stained dead cells. Scale bar: 50 µm.

With the capability of stabilizing GOx in the photothermal hyperthermia environment, we utilized the SGA for bacteria elimination in vitro to assess the synergistic effect. Benefiting from the optical transparency, the SERS diagnosis capabilities of SGA, and the superior stability of Ag@G in antibacterial H_2_O_2_ environment, the bacterial infection could be identified by in situ SERS diagnosis before GOx‐synergistic PTT as illustrated in **Figure** **4**a. Firstly, various films were attached on the colonies of *S. aureus* and *E. coli* on agarose culture medium, respectively. Then, the bacterial infections were detected by SERS spectroscopy through the SGA. After Raman spectrum analysis, bacterial infection status could be identified. Finally, identified bacterial infections were subjected to different therapies.

There were different peaks between *S. aureus* and *E. coli* in the dotted box region of Raman spectra (Figure 4b; Figure S15a,b, Supporting Information). *E. coli* exhibited two intrinsic Raman peaks at ≈650 and ≈730 cm^−1^ that were attributed to the bending vibration of COO^−^ and ring mode of adenine respectively, while *S. aureus* did not. It could be that *E. coli* has more adenine‐related molecules (adenosine monophosphate, FAD, NAD, DNA, etc.) outside the cell wall.^[^ ^29^ ^]^ Peaks at ≈1445 and ≈1500 cm^−1^ in the Raman spectra of *E. coli* attributed to the CH_2_ deformation of proteins and phenylalanine respectively, while peak at ≈1460 cm^−1^ in the Raman spectra of *S. aureus* attributed to CH_2_ bending vibration of saturated lipids. The results were consistent with previous report that the cell wall of Gram negative *E. coli* contained more proteins than the Gram positive *S. aureus*.^[^ ^29^ ^]^ Therefore, the differences in the Raman signals of *E. coli* and *S. aureus* originated from the metabolic differences between different bacterial strains. Relative peak intensity was evaluated by the ratio of *I*/*I*_G_, where *I* represented the intensity of the bacterial signal peak to be investigated and *I*_G_ represented the intensity of G band as an internal standard, to minimize potential influence from the inhomogeneity of the plasmonic Ag@G. According to peak position and relative peak intensity, infection types can be distinguished by principal component analysis (PCA) (Figure 4c). To verify the SERS diagnosis results, *S. aureus* and *E. coli* on SGA were characterized by SEM as in Figure S15c,d in the Supporting Information, and the results demonstrated good consistency and accuracy. Then, the infected agarose culture mediums were subjected to GOx‐synergistic PTT under 808 nm laser irradiation. The treatment effect was assessed by colony counting (Figure S16, Supporting Information), and the results demonstrated more significant and thorough bacterial killing effect of GOx‐synergistic PTT (99.9% for *S. aureus*, 99.5% for *E. coli*) than that of single enzymatic treatment (12.5% for *S. aureus*, 48.7% for *E. coli*) or PTT (94.2% for *S. aureus*, 89.8% for *E. coli*) (Figure 4d,e). The H‐SGA group, treated by hyperthermia before therapy, showed excellent synergistic bacterial killing effect (99.7% for *S. aureus*, 99.2% for *E. coli*) comparable to SGA, which also verified the stabilization of GOx in SGA. The morphologies of treated bacteria in different groups were characterized by SEM (Figure S17, Supporting Information) and TEM (Figure 4f). The cell membrane of pathogenic bacteria treated by SG suffered local collapse of structure accompanied by holes, while the pathogenic bacteria treated by single PTT (SA + laser) suffered extensive cell membrane collapse and cytoplasmic pyknosis. Under the GOx‐synergistic PTT of SGA, the pathogenic bacteria possessed a more incomplete membrane structure and less cytoplasm, and the bacteria attaching on SGA in Figure S18 in the Supporting Information also suffered extensive cell membrane collapse. The antibacterial mechanism was further explored by *o*‐nitrophenyl‐*β*‐D‐galactoside (ONPG) test (Figure S19, Supporting Information). The OD_420_ values of the treatment groups were higher than that of the blank group, indicating the release of *β*‐galactosidase from bacterial cells and destroy of bacterial cell membrane structure in the treatment groups. Comparing to the single enzymatic treatment and single PTT, the OD_420_ of the group treated by GOx‐synergistic PTT was higher, which demonstrated more *β*‐galactosidase was released and more membrane structure damage was caused in the process of GOx‐synergistic PTT. Bacterial cells were further subject to Live/Dead staining. The results demonstrated more dead bacteria killed by GOx‐synergistic PTT, and confirmed GOx‐synergistic PTT was a more efficient bactericidal strategy than single enzymatic treatment or PTT (Figure 4g,h).

**Figure 5:**
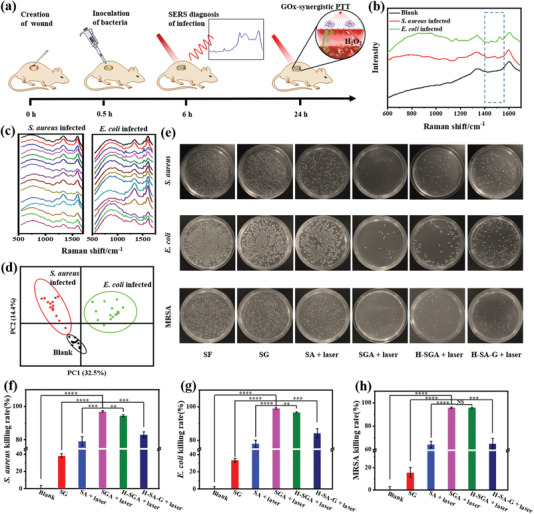
GOx‐synergistic PTT of bacterial infections in vivo. a) Schematic diagram illustrating GOx‐synergistic PTT of bacterial infections in vivo after SERS identification. b,c) Difference in SERS fingerprint spectra of *S. aureus* and *E. coli* in vivo. d) PCA identification of *S. aureus* and *E. coli* infections in vivo. e) Colony counting and f–h) quantification of bacterial colonies after different treatments in vivo. Error bar represents mean ± s.d., *n* = 3, ***p < 0.001, **p < 0.01, *p < 0.05 (two‐tailed *t*‐test). NS, not significantly different.

With excellent synergistic therapeutic effect for bacterial infections in vitro, SGA was further applied to the wound infection model on mice. Schematic diagram illustrating in vivo GOx‐synergistic PTT of bacterial infections was shown in **Figure** **5**a. Briefly, *S. aureus*, *E. coli* or MRSA were inoculated on wounds respectively. Following the inoculation, various films were attached on the wounds respectively. Then, the SGA group was subjected to SERS diagnosis to identify infection. After SERS identification, single enzymatic therapy of SG, single PTT of SA and GOx‐synergistic PTT of SGA, H‐SGA, and H‐SA‐G were proceeded to evaluate the effects of different therapies in vivo.

The SERS diagnosis results in Figure 5b,c demonstrated similar Raman peak differences as in vitro between *E. coli* and *S. aureus* in the right dotted box region of Figure 5b, which could be attributed to the good absorption of targeted molecules of bacteria on SGA. However, the Raman peaks of *E. coli*, presented in the left dotted box region of Figure 4b in vitro, were not present in Figure 5b in vivo. Such Raman spectra difference between in vitro and in vivo could originate from the nonspecific adsorption of interfering species from complex environment of wounds, which made the in vivo SERS identification challenging.^[^ ^30^ ^]^ With the superior SERS capability of Ag@G, infected bacteria types on wounds were also identified by means of PCA (Figure 5d). Moreover, besides pathogenic bacteria, SGA could also be able to detect bactericidal drugs administrated to wounds and monitor potential chemotherapy processes. As a model drug, bactericidal CV was detected on wounds (diameter of 10 mm) through SGA, and the results demonstrated an ultra‐low detection limit of 10 × 10^−9^ m, indicating the powerful capacity of SGA to monitor drugs in situ on wounds (Figure S20, Supporting Information). After SERS identification, the mice with infected wounds were subjected to GOx‐synergistic PTT (Figure S21, Supporting Information). The treatment results were assessed by colony counting (Figure 5e) and the statistical results were shown in Figure 5f–h. The SGA system exhibited 97.0% and 99.1% killing rate for *S. aureus* and *E. coli* respectively. Notably, the SGA system also exhibited superior bactericidal effect against MRSA with a killing rate of 95.8%. Based on the evidence above, the GOx‐synergistic PTT system of SGA demonstrated a more efficient bactericidal effect than single enzymatic treatment or PTT, which could effectively minimize the development of drug‐resistant bacteria infection and the deterioration of the wound.

**Figure 6:**
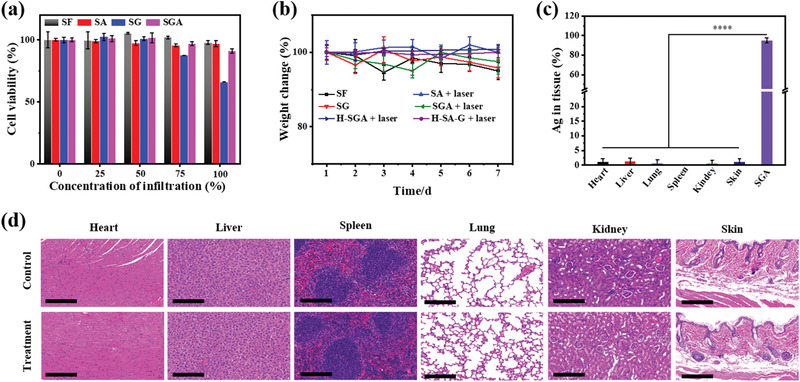
Biocompatibility of SGA. a) Mouse NIH/3T3 cell cytotoxicity of SGA. b) Weight changes of mice after different treatment. c) Ag@G distribution in various tissues. d) Histopathology analysis of various organs stained with H&E after GOx‐synergistic PTT. Scale bar: 200 µm.

Although the SGA possessed the capabilities of in situ SERS diagnosis and efficient GOx‐synergistic PTT effect, its biocompatibility still needs to be examined before practical applications. Mouse NIH/3T3 cell, a type of fibroblast closely involved in wound healing, was employed to assess the cytotoxicity of SGA (**Figure** **6**a). The results revealed a dose‐dependent toxicity from embedded GOx and the toxicity could be ignored below the concentration of 75% (GOx, 75 U L^−1^). AgNPs could accept electron from electron donors, such as graphene oxide, and obtain enhanced catalytic bactericidal capacity,^[^ ^31^ ^]^ which suggested that Ag@G might have potential long‐term cytotoxicity. Therefore, a 3 d cytotoxicity test of NIH/3T3 cell and a 5 d cytotoxicity test of C3H/10T1/2 cell were implemented using the SA (contain 128 µg Ag@G on SF) (Figure S22, Supporting Information) to investigate the long‐term cytotoxicity of Ag@G in the SGA system. The results revealed a negligible toxicity, which benefited from the good stability of SA and Ag@G. The weight changes of mice subjected to different treatments were recorded as in Figure 6b, and the weight of all mice were not less than 95% of their initial weight, suggesting their healthy state and the biosafety of SGA. The mice were sacrificed, and the distribution of Ag@G in various organs was studied by inductively coupled plasma mass spectrometry (ICP‐MS) (Figure 6c). The results demonstrated that almost all Ag@G remained on the SGA. Meanwhile, these organs were stained with Hematoxylin‐Eosin (H&E) (Figure 6d), and no obvious organ lesions caused by SGA were found. All these evidences confirmed the excellent biocompatibility of SGA.

## 3 Conclusion

In conclusion, we fabricated an efficient GOx‐synergistic PTT system integrating in situ bacterial SERS identification. The Ag@G showed superior stability in the H_2_O_2_‐rich environment due to the protection of graphitic shell, ensuring the robust SERS and photothermal conversion effect. Thermally unstable GOx was stabilized in SGA to obtain thermostability, facilitating the synergistic antibacterial effect of GOx in the hyperthermia environment of PTT. Benefiting from the optical transparency of SGA and plasmonic property of Ag@G, the intrinsic bacterial Raman signal in situ was obtained through the SGA. With in situ bacterial SERS identification and nonresistant GOx‐synergistic PTT effect, the SGA realized more efficient bactericidal effect on *E. coli*, *S. aureus* and MRSA in vivo than single PTT and enzymatic therapy, while without causing significant biotoxicity. This proposed GOx‐synergistic PTT system provides unstable therapeutic proteins with new opportunities in hyperthermal environment for efficiently eliminating bacterial infection.

## 4 Experimental Section

### Materials

Citric acid monohydrate, ethanediamine, glucose, sodium bicarbonate (NaHCO_3_), and nutrient agar were purchased from Sinopharm Chemical Reagent Co., Ltd (Shanghai, China). Luria‐Bertani (LB) broth medium was provided by Beijing AOBOX Bio‐Tech Co., Ltd (Beijing, China). Fumed silica, silver nitrate (AgNO_3_), lithium bromide (LiBr), dipicolinic acid (DPA), and crystal violet (CV) were obtained from Aladdin (Shanghai, China). *Bombyx mori* silkworm cocoons were purchased from Guangxi Sericulture Technology Co., Ltd (Guangxi, China). Horse radish peroxidase (HRP; >300 U mg^−1^), glucose oxidase (GOx; >100 U mg^−1^) and 3,3′,5,5′‐tetramthylbenzidine (TMB) were provided by Sigma‐Aldrich (Shanghai, China). Gram‐negative *Escherichia coli* (*E. coli*, *ATCC 25922*), gram‐positive *Staphylococcus aureus* (*S. aureus*, *ATCC 6538*) and methicillin‐resistant *Staphylococcus aureus* (MRSA, *ATCC 43300*) were purchased from Shanghai Luwei Science and Technology Co., Ltd. (Shanghai, China). Dulbecco's modified Eagle medium (DMEM), trypsin, penicillin‐streptomycin (PS), fetal bovine serum (FBS) and acridine orange/propidium iodide (AO/PI) were purchased from Thermo Fisher Scientific (USA). The mouse embryonic fibroblasts NIH/3T3 and mouse fibroblasts C3H/10T1/2 were obtained from Cell Bank of Chinese Academy of Sciences (Shanghai, China). The BALB/c female mice (8 weeks old) were obtained from Hunan SJA Laboratory Animal Co., Ltd (Hunan, China), and all mice received care under protocols approved by the Institutional Animal Care and Use Committee of Hunan University. Deionized water with a specific resistance over 18.2 MΩ cm^−1^ was employed throughout the experiment.

### Synthesis of Ag Graphitic Nanocapsule (Ag@G)

Before the synthesis of Ag@G, a N‐doped carbon quantum dot (NCQD) precursor was prepared based on the previous reports.^[^ ^32^ ^]^ First, 1.149 g citric acid monohydrate and 335 µL ethanediamine were successively dissolved into 40 mL deionized water. Then the mixture was put into a hydrothermal synthesis reactor and heated in an oven at 150 °C for 6 h to prepare NCQD. The prepared NCQD was stored at room temperature. Ag@G was synthesized by a chemical vapor deposition (CVD) process.^[^ ^22^, ^33^ ^]^ Simply, 1 g fumed SiO_2_ was dispersed into 80 mL methanol solution. After ultrasound for 1 h, 67 mg AgNO_3_ and 10 µL NCQD solution were added into the mixed solution. After ultrasound for another 2 h, the mixed solution was evaporated to obtain the catalyst powder. Finally, the powder was ground evenly and put into a tube furnace to grow Ag@G at 800 °C for 10 min with a H_2_ flow rate of 10 sccm. After HF‐etching, cleaning and centrifugation of the mixed powder, the Ag@G nanoparticles were obtained. Bare Ag nanoparticle (AgNP) was synthesized through the method reported previously.^[^ ^34^ ^]^ The prepared Ag@G and AgNP were also stored at room temperature.

### Fabrication of Silk‐GOx‐Ag@G (SGA) Complex Film

SGA was fabricated by mixing GOx and regenerated silk fibroin (RSF) and spreading the mixed glue into a film. Briefly, RSF was prepared using a previously reported method through series of processes of silk degumming, dissolution and dialysis.^[^ ^35^ ^]^ First, 10 g natural cocoons were added into 200 mL 0.5 wt% NaHCO_3_ aqueous solution which was subsequently boiled for 0.5 h and washed for three times. After the boiling and washing process was repeated for three times, the degummed silk was obtained and dried for later use. Then 1 g of degummed silk was cut into pieces and added into 10 mL 9.3 m LiBr solution, and the mixed solution was heated in an oven at 60 °C for 1 h under continuous stirring to dissociate silk fibroin.^[^ ^36^ ^]^ Next, the dissociated solution was put into a dialysis bag (3 KD) which was put into deionized water for 12 h (deionized water was changed every 3 h) to remove undesired small molecules. After RSF was obtained, a small amount of RSF was taken out to dry and weighed to calibrate the concentration of RSF, and the prepared RSF was stored in a refrigerator at 4 °C. Finally, pure silk film (SF) and silk films with different enzyme contents (SF‐GOx, SG) were spread out after mixing RSF and GOx at different weight ratios. Whereafter, these SF and SG were put into the microarray dot system (Crysta Core Smartarrayer 48) to spray Ag@G dot matrix to prepare SF‐Ag@G (SA) and SF‐GOx‐Ag@G (SGA) of different specifications.

### Morphology and Structure Characterization of Ag@G/SGA

1 mL Ag@G aqueous solution was used to test the UV–vis absorption spectrum of Ag@G by UV–vis spectrometer (UV‐2600i, Shimadzu, Japan), and the size distribution and surface charge of Ag@G were tested on the dynamic light scattering (DLS) instrument (Nano‐ZS90, Malvern, UK). After 8 µL Ag@G aqueous solution was dropped onto the formvar/carbon supported copper grid and dried, the morphology of Ag@G was characterized by transmission electron microscopy (TEM) (JEM‐2100plus, JEOL, Japan). SF, SG, SA, and SGA of different specifications were subjected to UV–vis spectrometer to collect transmittance spectra, to infrared spectrometer (FTIR 6700, Nicolet, USA) to collect infrared spectra and to Raman spectrometer (inVia‐Reflex, Renishaw, UK) to collect Raman spectra, then their hydrophilic performances were evaluated on the contact angle measurement instrument (JC2000D2 (RT‐400), POWEREACH, China) and their mechanical properties were tested on the tensile tester (HYC‐2011, HONGJIN, China). Finally, the morphology of SGA was observed by scanning electron microscopy (SEM) (JSM‐6700F, JEOL, Japan).

### SERS Effect Evaluation of Plasmonic SGA

Raman signal spectra of 100 × 10^−6^ m disinfectant crystal violet (CV) aqueous solution and the mixed aqueous solution of 1 × 10^−6^ m CV and 700 µg mL^−1^ Ag@G were collected using Raman spectrometer to evaluate the Raman enhancement effect of Ag@G. Then, 50 µL CV of different concentrations (100 × 10^−12^ m, 1 × 10^−9^ m, 10 × 10^−9^ m, 100 × 10^−9^ m, 1 × 10^−6^ m) were dropped on SGA (4 mg mL^−1^ Ag@G) and dried, and their Raman imagings through the films were collected to evaluate the Raman detection sensitivity of SGA.

### Photothermal Effect Evaluation of Plasmonic SGA

The photothermal conversion of SGA with different Ag@G (2.5 mg mL^−1^) spraying times was investigated by infrared thermal imaging camera (FOTRIC 365). The power of 808 nm laser was 3 W cm^−2^. To demonstrate the excellent thermal stability of SGA, the photothermal cycling of the SGA with excessive Ag@G (4 mg mL^−1^, sprayed for 4 times) was evaluated by a 5 W cm^−2^ 808 nm laser.

### Bacterial Culture

Gram‐negative *E. coli*, gram‐positive *S. aureus* and MRSA were inoculated into nutrient agar by streak plate method. After cultured in a constant temperature incubator (HF‐240, HEAL FORCE) for 24 h, colonies were transferred to 25 mL sterile Luria‐Bertani (LB) broth medium at 37 °C and cultured to mid‐log phase. After centrifugation and washing for three times, bacterial suspensions with different concentration in PBS were prepared. The concentrations of bacterial suspensions were estimated by flat colony counting method.

### Measurement of Enzyme Activity

Enzyme activities of GOx in SG and free GOx were assessed by employing glucose as the substrate and monitoring the oxidation rate of TMB. Firstly, the GOx in SG (RSF/GOx weight ratio: 10, 50, 200) was released to 1mL phosphate buffer before assessment. Then, 1 mL phosphate buffer (0.1 m, pH = 6.5) containing 150 × 10^−6^ m glucose, 250 × 10^−6^ m TMB, 1.25 × 10^−6^ wt% horseradish peroxidase (HRP) and theoretical 0.5 U GOx from SG or free GOx treated with different temperatures for 7 d was immediately put into the UV–vis spectrometer to collect the catalytic kinetics curves at 25 °C. During the collection, absorbance at 635 nm was recorded continuously for 560 s and the absorbance curve was plotted versus time. ΔA_635_ at 336 s from the linear region of the kinetics curve was assumed as enzymic activity, and enzymic relative residual activity of SG was defined as ΔA_635(SG)_/ΔA_635(GOx)_. Then, the enzyme activity of SG was evaluated by its inhibitory effect on *E. coli* and *S. aureus*. After treated at different temperatures for 7 d, SG (RSF/GOx weight ratio: 500, 2000, 10 000) and FP‐GOx (GOx‐loaded filter paper, FG) were attached on the nutrient agar medium with the same inoculation number of bacteria, and the diameter D value of inhibition zone was compared to evaluate the enzyme activity. In order to evaluate the GOx stabilization effect of SGA (with GOx inside) and SA‐G (with GOx coated on surface), SGA and SA‐G were subjected to photothermal treatment at 85 °C for 1 h to obtain H‐SGA and H‐SA‐G. Similarly, the inhibition zone diameters of SA, SGA, H‐SGA, and H‐SA‐G were further compared. The bacterial culture medium used above was supplemented with 5 × 10^−3^ m glucose. The free GOx, SG, SGA, H‐SGA were subjected to enzyme kinetics test to calculate the Michaelis constant *K_m_* by Lineweaver‐Burk plots.

### Cell Toxicity of SGA

The mouse embryonic fibroblasts (NIH/3T3) were cultured in DMEM culture medium supplemented with 1% PS and 10% FBS solution in an incubator (37 °C, 5% CO_2_). Cell Counting Kit‐8 (CCK‐8) was employed to measure the cell viability of NIH/3T3 cells. First, the infiltrations of SF, SA, SG and SGA (diamter of 20 mm) were extracted through immersing these films in 10 mL DMEM at 37 °C for 24 h respectively. Meanwhile, cells with 100 µL culture medium were seeded into 96‐well plates (10 000 cells each well) and incubated for 24 h to permit cell adhesion. Then, the infiltrations of SF, SA, SG, and SGA were diluted with DMEM to concentrations of 25%, 50%, 75%, 100% respectively. Next, cells were maintained in 100 µL culture medium with or without four different diluted infiltrations for 24 h. Finally, 100 µL fresh cell culture medium containing 10 µL CCK‐8 was added to each well after removal of the supernatant, and the plates were incubated for another 1 h in a dark incubator. The absorbance at 450 nm was measured by a multi‐detection microplate reader (Synergy2, Bio‐Tek, USA) and the assay was repeated for three times. Similarly, The NIH/3T3 cell was continuously incubated for 3 d with SF and SA infiltration respectively, and the C3H/10T1/2 cell was continuously incubated for 5 d with SF and SA infiltration respectively to investigate the long‐term toxicity of Ag@G on SF.

### In Vitro SERS Diagnosis of Bacterial Infection

2 µL 2 × 10^5^ CFU *E. coli* and *S. aureus* cells were respectively inoculated on the nutrient agar (containing 5 × 10^−3^ m glucose) in the 96‐well plate and incubated for 6 h at 37 °C (5% CO_2_). Then, SF, SG, SA, SGA, H‐SGA, H‐SA‐G (all 1 cm^2^) were covered on the nutrient agar. After incubated for 0.5 h in the bacterial incubator, the SGA group were subject to SERS diagnosis in situ. The SERS spectra were assessed by principal component analysis (PCA) to identify different bacterial infections.

### In Vitro Synergistic PTT for Bacterial Infection

After bacterial identification by SERS diagnosis, SA, SGA, H‐SGA, H‐SA‐G groups were subject a GOx‐synergistic PTT process at 60 °C for 5 min with an 808 nm laser. Then the samples in the wells were respectively dispersed into 5 mL water, and 50 µL dispersing solution was taken out for bacterial growth in plate. After 1 d incubation, the colony count was carried out and the synergistic bactericidal effect was evaluated. GOx‐synergistic PTT effect was also evaluated in bacterial dispersion liquid. SF, SG, SA and SGA were added into 1 mL PBS containing 1 × 10^11^ CFU bacteria and 5 × 10^−3^ m glucose, respectively. After incubation for 0.5 h, the SA group and SGA group were irradiated by laser, and the photothermal temperature was maintained at 60 °C for 2 h. Then, all films were removed, the liquid samples of all groups were centrifuged to isolate bacteria and divided into two parts. One was stained with AO/PI (Acridine Orange/Propidium Iodide), living/dead bacteria were observed at 530/630 nm emission with 488 nm excitation under a fluorescent confocal microscope (FCM) to evaluate the bactericidal effect. Another portion was immobilized with 25% glutaraldehyde aqueous solution for 2 h, and subsequently dehydrated and cleaned with gradient ethanol of 25%, 50%, 75%, and 100%. Then 8 µL ethanol solution of immobilized bacteria was taken out and added to the formvar/carbon supported copper grid. After the copper grid was dried, bacterial morphology was observed by SEM to evaluate the bactericidal mechanism. The antibacterial mechanism was also explored by o‐nitrophenyl‐*β*‐D‐galactoside (ONPG) test. After different treatments, the groups of blank, SG, SA + laser and SGA + laser were incubated with 2 × 10^−3^ m ONPG for 10 min at 37 °C. Then, the OD_420_ values of the supernate from these groups were tested.

### In Vivo Raman Diagnosis of Bacterial Infection

First, a full‐thickness cutaneous wound model (diamter of 3 mm) was established on the back of BALB/c female mice (8 weeks old). Then, 2 µL bacterial solution containing 2 × 10^8^ CFU bacterial cells (*E. coli*, *S. aureus*, MRSA) was distributed onto the wounds of mice, and SF, SG, SA, SGA, H‐SGA, H‐SA‐G were attached to the wounds respectively. After the mice were housed for 5.5 h, the SERS spectra of SGA group with different bacterial infections and blank group were collected on Raman spectrometer. Then, the SERS spectra were assessed by PCA to identify the two types of infection.

### In Vivo Synergistic Treatment for Bacterial Infection

After all groups of mice were fed for 24 h, the SA, SGA, H‐SGA, and H‐SA‐G groups were irradiated with 808 nm laser at 60 °C for 3 min, and the laser power was 1.5 W cm^−2^. Then, the samples from wounds were harvested and dispersed into PBS. Subsequently, the bactericidal effect was assessed by plate colony count.

Calculation of the Degree of Synergism: The extent of synergism was quantitatively expressed as “degree of synergism” (DS) in co‐catalytic process of mixed enzymes.^[^ ^37^ ^]^ The value of DS was equal to the ratio of the practical activity exhibited by mixtures divided by the sum of the activities of separate components. Based on this definition, the DS of antibacterial effect was calculated in the SGA system by the ratio of the antibacterial effect exhibited by SGA divided by the sum of the antibacterial effect of single enzyme and single PTT.

### Biocompatibility of SGA

The above experimental groups were continuously fed for a week, and the body weights of mice were recorded every day. The biocompatibility of the silk composite films was evaluated by comparing the body weight changes of mice. Mice in the SGA group and the blank group were sacrificed after one week of culture, and the SGA on wound, internal organs (heart, liver, spleen, lung, and kidney) and part of the skin adjacent to wound were taken out to be dissolved in concentrated nitric acid by water bath heating. After the dissolution of concentrated nitric acid, the Ag@G in different groups was dissolved into Ag^+^. Then, the contents of Ag^+^ in different groups were quantitatively analyzed by inductively coupled plasma mass spectrometry (ICP‐MS). By comparing the normalized Ag^+^ contents (per unit tissue weight) in different groups, the biological distribution of Ag@G and the stability of SGA were evaluated. Meanwhile, the skin and internal organs were stained with hematoxylin‐eosin (H&E) and made into slices. The morphology of the organ cells was observed with a section scanner (Pannoramic MIDI, 3DHISTECH, Hungary) to evaluate the biotoxicity of SGA.

### Statistical Analysis

First, average value and standard deviation (s.d.) of the results were calculated. Then, two‐tailed *t*‐test was employed to evaluate the statistical significant difference between groups by GraphPad Prism 6. Results were presented with mean ± s.d. and the significance levels were *p < 0.05, **p < 0.01 and ***p < 0.001, *p* > 0.05, NS was considered insignificant.

## Conflict of Interest

The authors declare no conflict of interest.

## Supporting information

Supporting Information

## Data Availability

The data that support the findings of this study are available from the corresponding author upon reasonable request.
