# Supplementary material for: Stabilizing Enzymes in Plasmonic Silk Film for Synergistic Therapy of In Situ SERS Identified Bacteria
Source: Adv Sci (Weinh). 2022 Jan 6;9(6):2104576. doi: 10.1002/advs.202104576 (PMC8867187; doi:10.1002/advs.202104576)
Supplement: Supplementary file 1 — Supporting Information [file ADVS-9-2104576-s001.pdf]

WILEY-VCH

ADVANCED SCIENCE
Open Access

Supporting Information

for *Adv. Sci.*, DOI: 10.1002/advs.202104576

Stabilizing Enzymes in Plasmonic Silk Film for Synergistic Therapy of In Situ SERS Identified Bacteria

*Zhangkun Liu, Shengkai Li, Zhiwei Yin, Zhaotian Zhu, Long Chen, Weihong Tan, and Zhuo Chen**

Supporting Information

Stabilizing Enzymes in Plasmonic Silk Film for Synergistic Therapy of In Situ SERS Identified Bacteria

*Zhangkun Liu, Shengkai Li, Zhiwei Yin, Zhaotian Zhu, Long Chen, Weihong Tan, and Zhuo Chen**

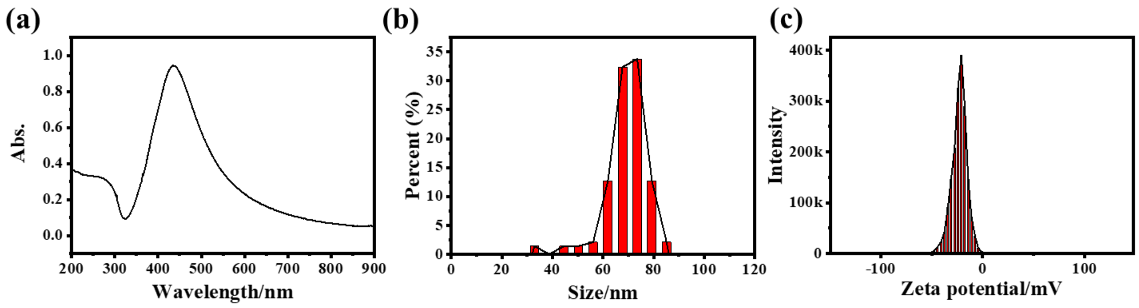

Figure S1 (a) UV-vis absorption spectrum of Ag@G. (b) Size distribution of Ag@G. (c) Zeta potential of Ag@G.

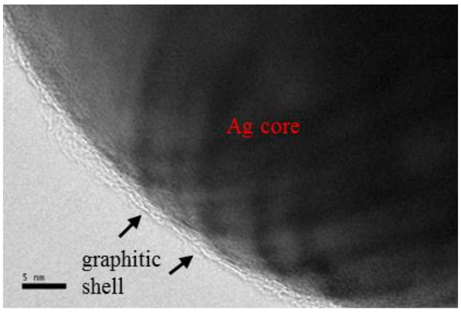

Figure S2 Enlarged TEM image of Ag@G with core-shell structure.

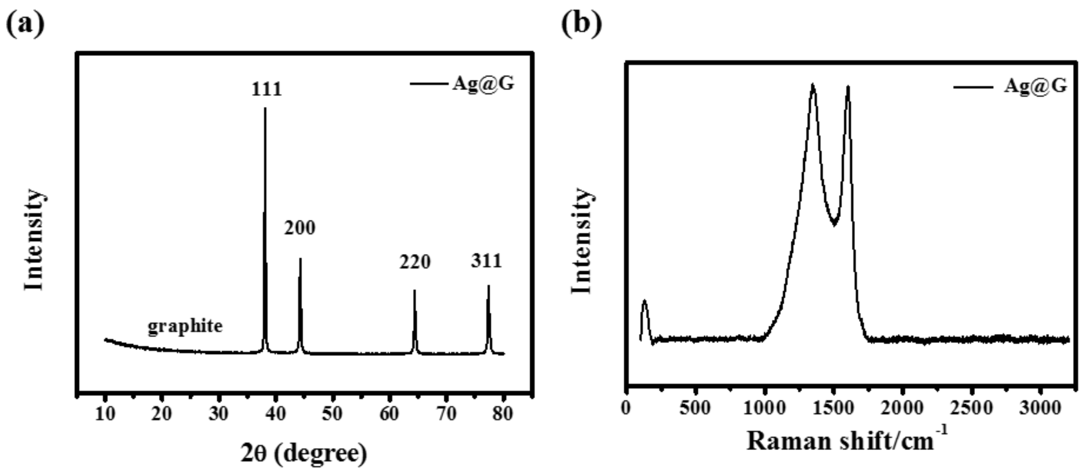

Figure S3 (a) XRD and (b) Raman spectra of Ag@G.

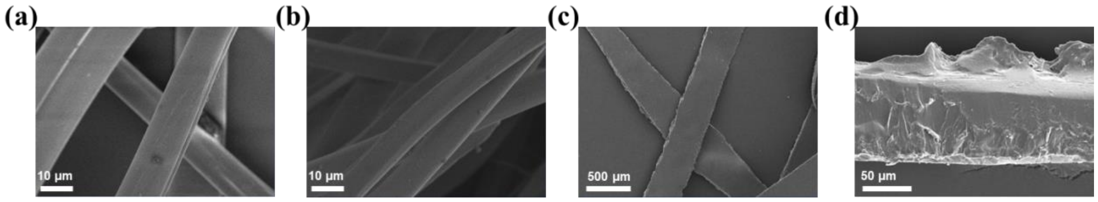

Figure S4 SEM images of silk (a), degummed silk (b), surface (c) and section (d) of SF.

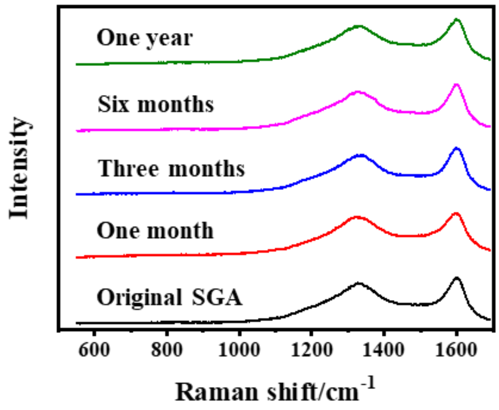

Figure S5 SERS spectra of SGA stored at room temperature for various time.

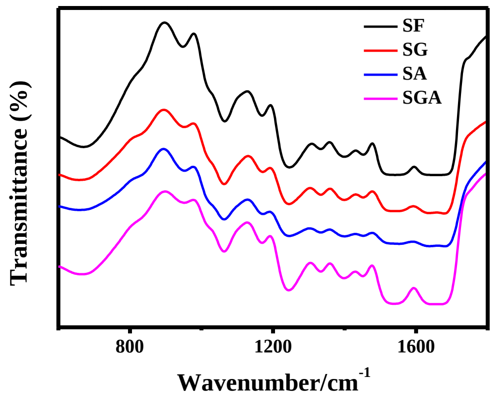

Figure S6 FTIR spectra of SF, SG, SA and SGA.

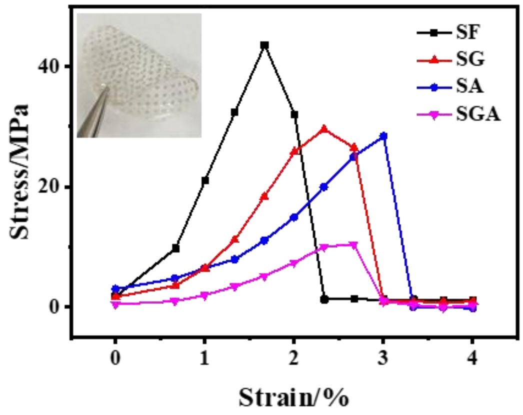

Figure S7 Mechanical property of SF, SA, SG and SGA.

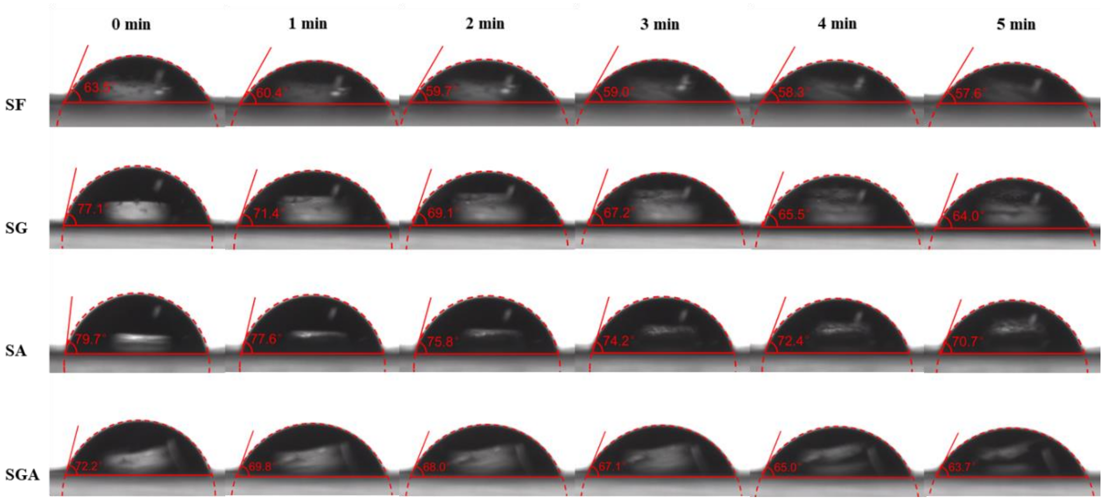

Figure S8 Hydrophilic performance of SF, SG, SA and SGA.

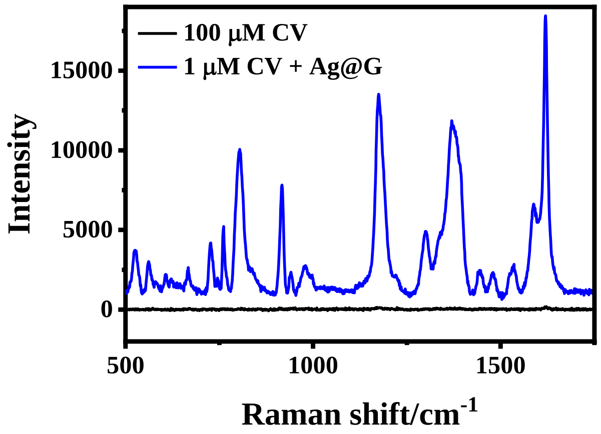

Figure S9 SERS detection for disinfectant CV *in vitro* by 700 mg/L Ag@G.

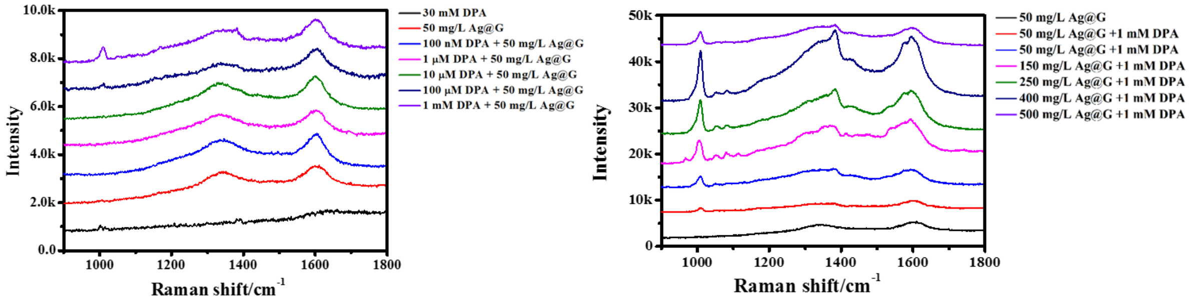

Figure S10 SERS detection for dipicolinic acid (DPA) from bacterial spore *in vitro* by Ag@G.

Raman EF Evalution of plasmonic SGA

Simply, we estimated the EF by the following equation:

$$EF=\frac{I_{SERS}/C_{SERS}}{I_{RS}/C_{RS}}$$

I_{SERS} represents the Raman spectrum intensity at 1175 cm^{-1} of CV with a concentration C_{SERS} in the presence of Ag@G while I_{RS} represents the intensity of CV with concentration C_{RS} without Ag@G. I_{SERS} and I_{RS} were measured under identical conditions and the detailed calculation is shown below.

(1) The C_{SERS} was 1×10^{-6} M, the obtained I_{SERS} was ~ 12520 counts/s.

(2) The C_{RS} was 1×10^{-4} M, the measured I_{RS} was ~ 113 counts/s.

(3) the analytical EF $\approx 1.1 \times 10^{4}$.

Similarly, the EF of dipicolinic acid (DPA) was also estimated by the equation.

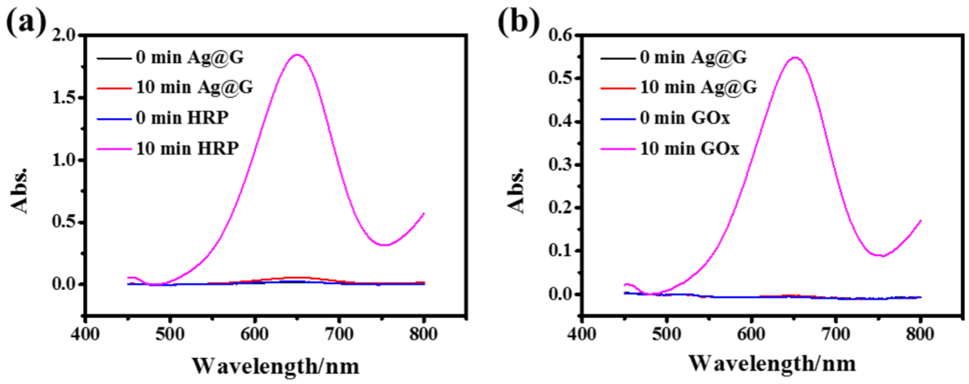

Figure S11 enzyme-like activity test. (a) Catalytic activity of HRP (0.025 ppm) and Ag@G (1 mg/L) with 15 μM glucose and 25 μM TMB. (b) Catalytic activity of GOx (0.5 U/ml) and Ag@G (1 mg/L) with 15 μM glucose, 25 μM TMB and 0.025 ppm HRP.

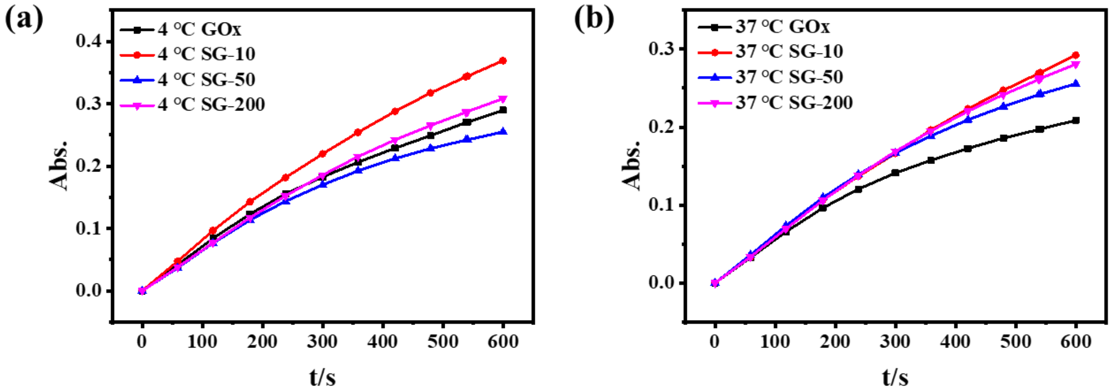

Figure S12 Catalytic kinetics of GOx stabilized in SG with different ratios (fibroin/GOx) after 4 °C (a) and 37 °C (b) treatment for 7 d.

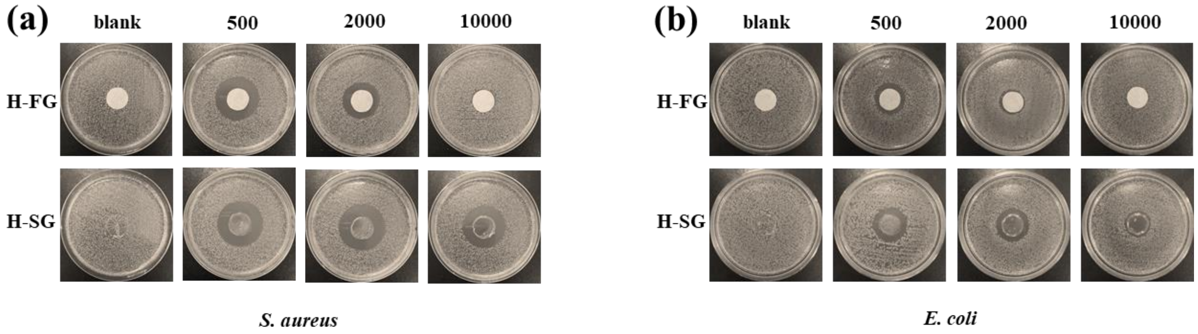

Figure S13 Antibacterial activity of FG and SG with different ratios (fibroin/GOx) after 25 °C treatment for 7 d for *S. aureus* (a) and *E. coli* (b).

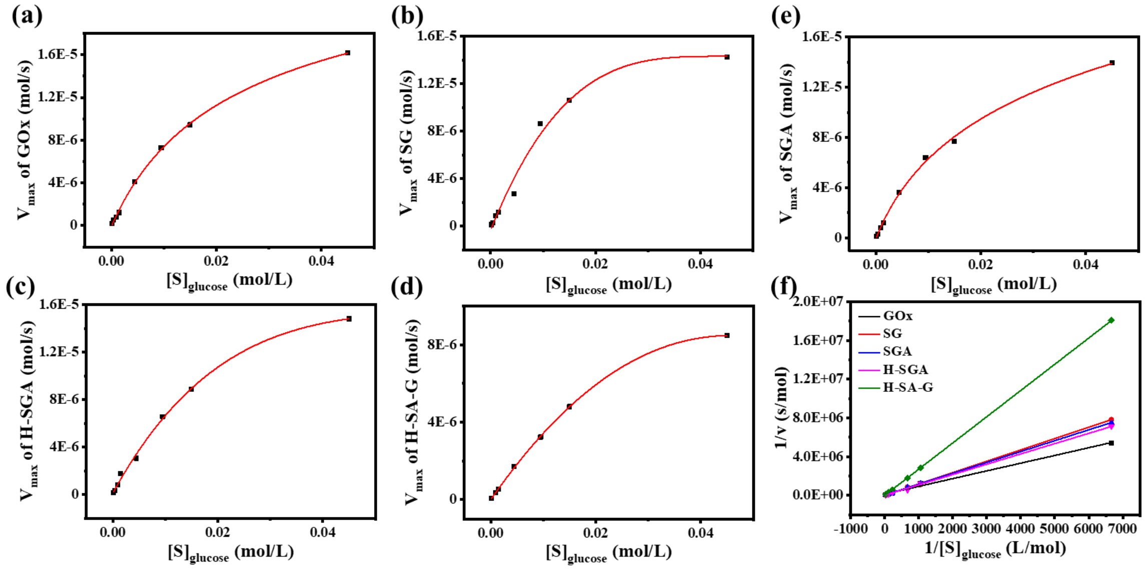

Figure S14 Enzyme kinetics tests of GOx, SG, SGA, H-SGA and H-SA-G.

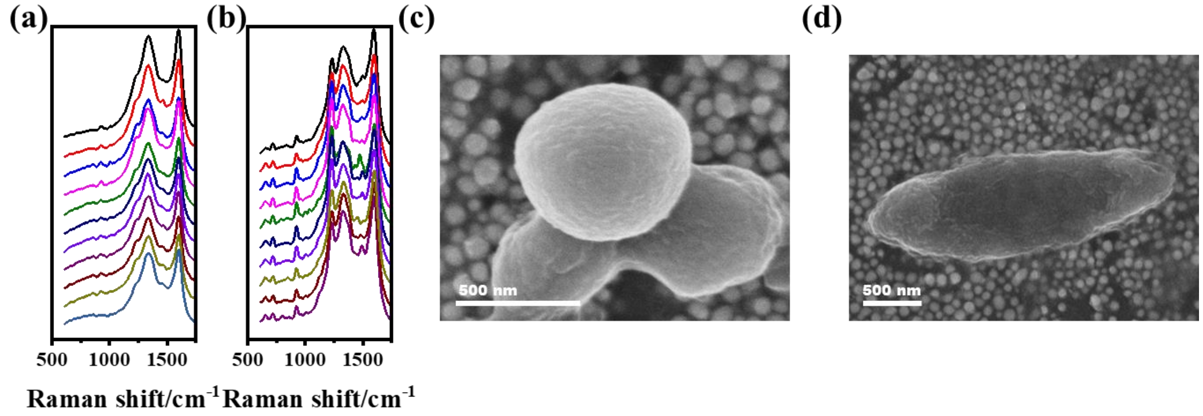

Figure S15 Raman fingerprint spectra of *S. aureus* (a) and *E. coli* (b) on SGA and SEM images of *S. aureus* (c) and *E. coli* (d) on SGA.

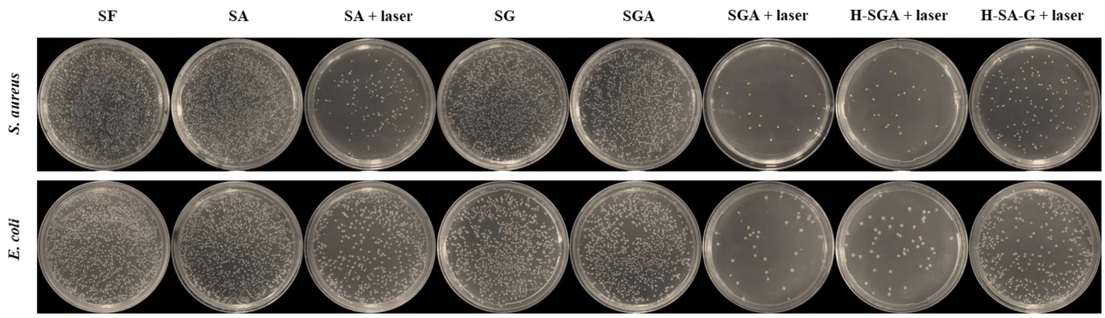

Figure S16 Bactericidal effect of different treatment groups on *S. aureus* and *E. coli* under the irradiation of 808 nm laser (3 W/cm^2).

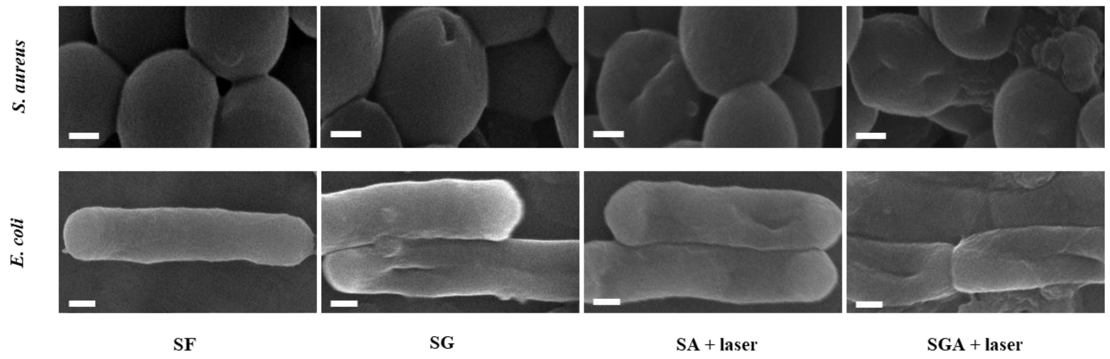

Figure S17 SEM images of synergetic killing for *S. aureus* and *E. coli in vitro* in different treatment groups under the irradiation of 808 nm laser (3 W/cm^2). Scale bar: 200 nm.

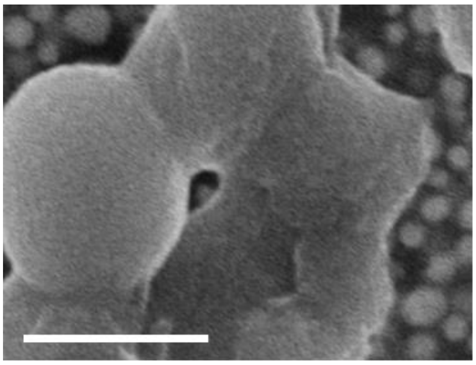

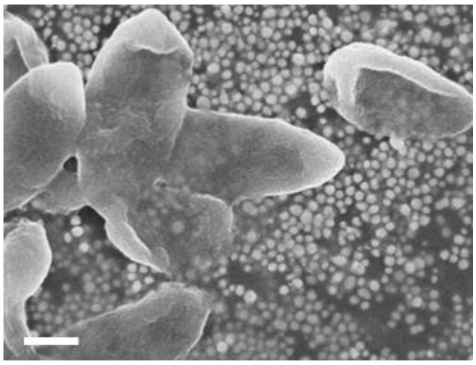

Figure S18 SEM images of GOx-synergetic photothermal killing for *S. aureus* and *E. coli* on SGA under the irradiation of 808 nm laser (3 W/cm^2). Scale bar: 500 nm.

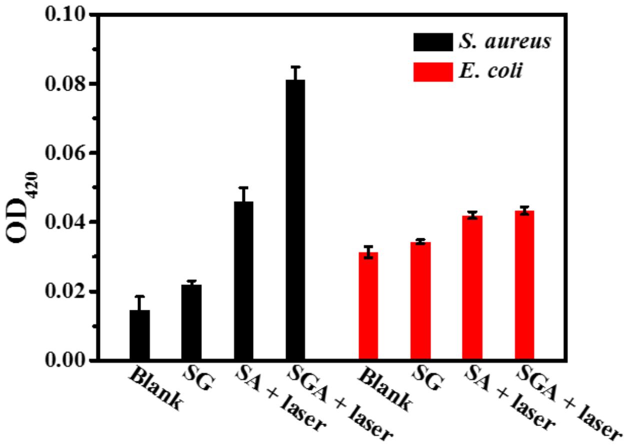

Figure S19 ONPG tests. O-nitrophenol absorbance at 420 nm of *S. aureus* and *E. coli* with different treatment.

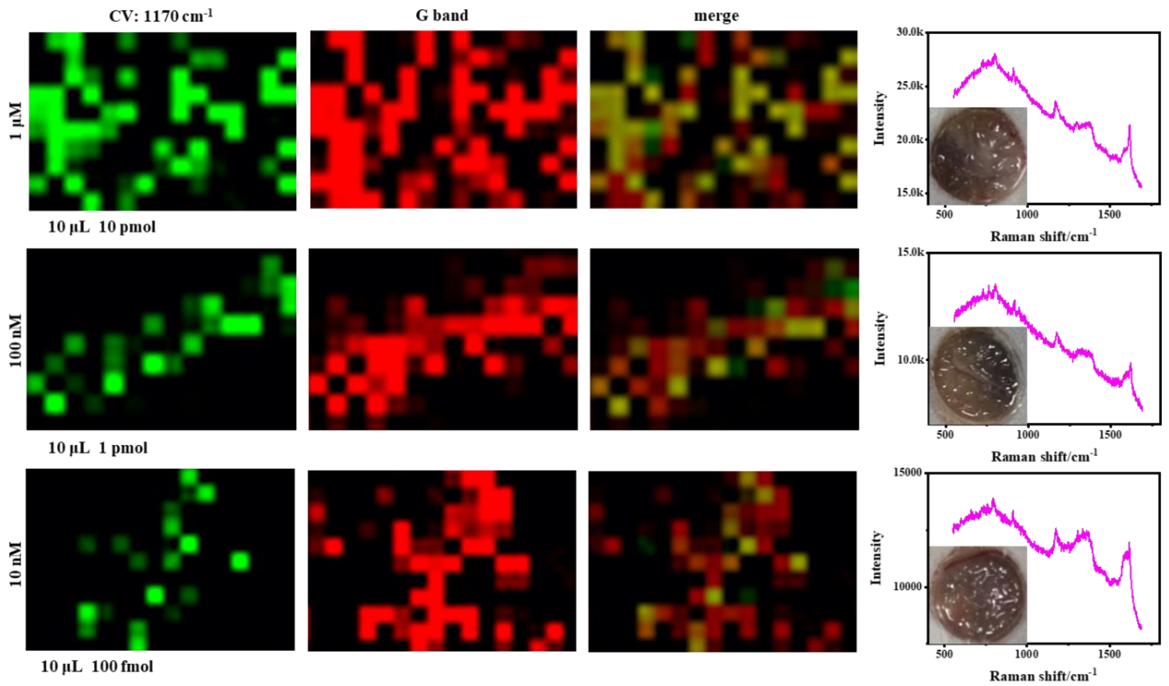

Figure S20 SERS imaging for disinfectant CV of different concentrations *in vivo* by SGA. Pixel: 2 μm × 2 μm.

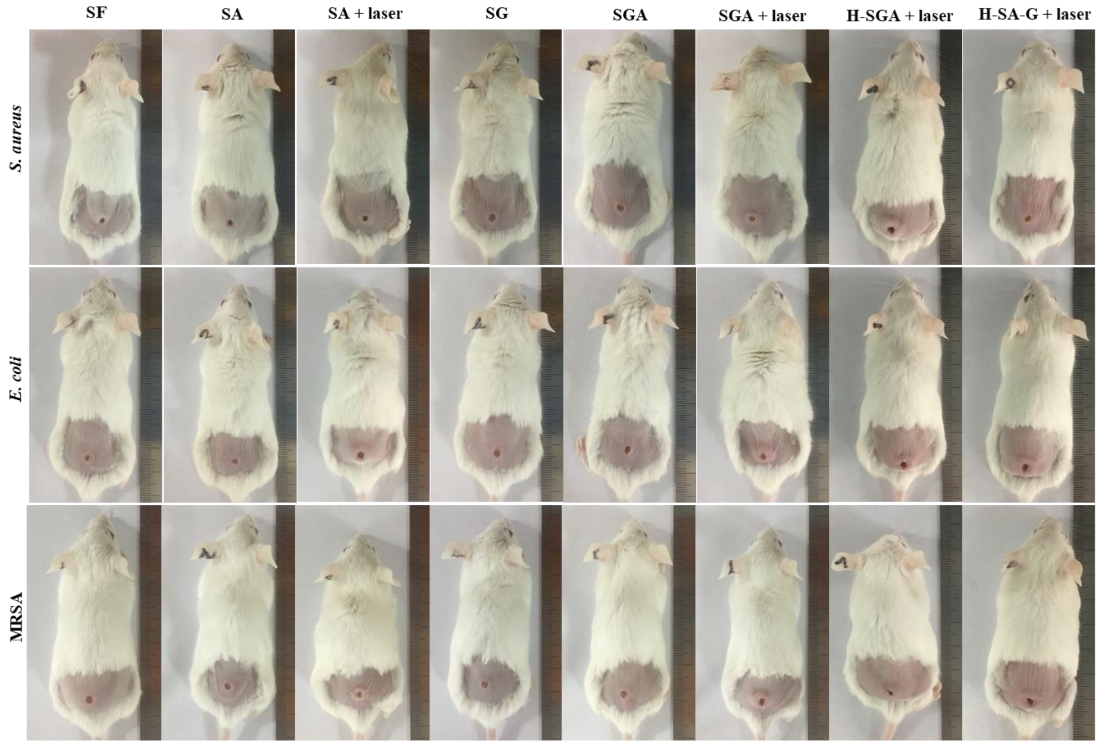

Figure S21 Photographs of bacteria-infected mice with different treatments.

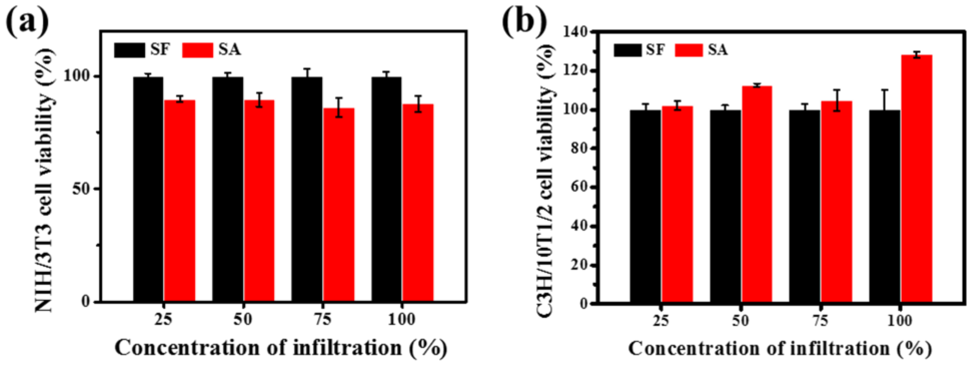

Figure S22 (a) Mouse NIH/3T3 cell cytotoxicity test after 3 d and (b) C3H/10T1/2 cell cytotoxicity test after 5 d.
